# A rehabilitation intervention to improve Recovery after an Episode of Delirium in adults over 65 years (RecoverED): a multi‐centre, single arm feasibility study

**DOI:** 10.1002/alz70860_097411

**Published:** 2025-12-23

**Authors:** Louise M Allan, Jinpil Um, Abby O'Connell, James Connors, Abigail Laverick, Alison Bingham, Kirstie Chandler, Shruti Raghuraman, Liz Goodwin, Linda Clare

**Affiliations:** ^1^ University of Exeter, Exeter, Devon, United Kingdom; ^2^ University of Exeter Medical School, Exeter, United Kingdom

## Abstract

**Background:**

Delirium, a common cognitive disorder in hospitalised older adults, prolongs stays and increases costs. However, research on post‐discharge rehabilitation interventions is limited despite its importance. This study examined the feasibility of a home‐based rehabilitation intervention designed to enhance recovery outcomes for older individuals after hospital discharge following delirium, aiming to inform the design of a future randomised controlled trial (RCT).

**Method:**

This study used a mixed‐methods design with an embedded process evaluation (reported separately) to assess the intervention's feasibility, fidelity, and acceptability. Participants over 65, diagnosed with delirium during hospitalisation, were recruited from six NHS hospitals across the UK. The intervention started within two weeks of discharge and consisted of personalised rehabilitation sessions delivered by healthcare professionals. Feasibility outcomes included recruitment, retention, adherence rates, and participant acceptability, with data collected through standardised measures.

**Result:**

In total, 419 patients were identified as having delirium and 36 met the full eligibility. Nineteen patient and carer pairs agreed to participate in the study (consent rate 53% (95% CI: 35%, 70%) with 13 participants going on to start the intervention (68% (95% CI: 43%, 87%) and 10 participants completing final follow‐up (53% (95% CI: 29%, 76%). Initial assessments occurred an average of 18 days (SD = 13.01) post‐discharge, with 77% completed within 14 days. Participants averaged eight sessions (SD = 2.9). 19 participants completed the primary outcome at baseline, while 10 participants completed it at 6‐month follow‐up. The economic evaluation indicated a total cost of £1,249.29 per participant, covering assessments, intervention sessions, and training costs.

**Conclusion:**

The RecoverED intervention showed feasibility among older adults recovering from delirium, as evidenced by the trial processes for participants who entered the study. However, recruitment challenges indicate a need for better strategies, and further research through a definitive RCT to demonstrate the effectiveness and cost‐effectiveness of the intervention.

